# Supplementary material for: Treadmill exercise modulates the medial prefrontal-amygdala neural circuit to improve the resilience against chronic restraint stress
Source: Commun Biol. 2023 Jun 9;6:624. doi: 10.1038/s42003-023-05003-w (PMC10256706; doi:10.1038/s42003-023-05003-w)
Supplement: Supplementary file 2 — Description of Additional Supplementary Files [file 42003_2023_5003_MOESM2_ESM.pdf]

Description of Additional Supplementary Files

File name: Supplementary Data

Description: All source data supporting the conclusion of this work
