# Supplementary material for: Treadmill exercise modulates the medial prefrontal-amygdala neural circuit to improve the resilience against chronic restraint stress
Source: Commun Biol. 2023 Jun 9;6:624. doi: 10.1038/s42003-023-05003-w (PMC10256706; doi:10.1038/s42003-023-05003-w)
Supplement: Supplementary file 4 — Reporting Summary [file 42003_2023_5003_MOESM4_ESM.pdf]

nature portfolio

Corresponding author(s): Kwok-Fai So, and Li Zhang

Last updated by author(s): May 31, 2023

Reporting Summary

Nature Portfolio wishes to improve the reproducibility of the work that we publish. This form provides structure for consistency and transparency in reporting. For further information on Nature Portfolio policies, see our Editorial Policies and the Editorial Policy Checklist.

Statistics

For all statistical analyses, confirm that the following items are present in the figure legend, table legend, main text, or Methods section.

n/a	Confirmed	
☐	☒	The exact sample size (*n*) for each experimental group/condition, given as a discrete number and unit of measurement
☐	☒	A statement on whether measurements were taken from distinct samples or whether the same sample was measured repeatedly
☐	☒	The statistical test(s) used AND whether they are one- or two-sided<br>*Only common tests should be described solely by name; describe more complex techniques in the Methods section.*
☒	☐	A description of all covariates tested
☐	☒	A description of any assumptions or corrections, such as tests of normality and adjustment for multiple comparisons
☐	☒	A full description of the statistical parameters including central tendency (e.g. means) or other basic estimates (e.g. regression coefficient) AND variation (e.g. standard deviation) or associated estimates of uncertainty (e.g. confidence intervals)
☐	☒	For null hypothesis testing, the test statistic (e.g. *F*, *t*, *r*) with confidence intervals, effect sizes, degrees of freedom and *P* value noted<br>*Give P values as exact values whenever suitable.*
☒	☐	For Bayesian analysis, information on the choice of priors and Markov chain Monte Carlo settings
☒	☐	For hierarchical and complex designs, identification of the appropriate level for tests and full reporting of outcomes
☒	☐	Estimates of effect sizes (e.g. Cohen's *d*, Pearson's *r*), indicating how they were calculated

Our web collection on statistics for biologists contains articles on many of the points above.

Software and code

Policy information about availability of computer code

Data collection	Zen (v8.1.0.484, Zeiss Inc.), Bio-Rad CFX Maestro (v1.0, BIO-RAD Inc.)
Data analysis	Excel (in Microsoft 365 package), Prism (v8.2, GraphPad); ImageJ (v1.52, NIH); R package edgeR; Clampfit (v10.0, Molecular Devices)

For manuscripts utilizing custom algorithms or software that are central to the research but not yet described in published literature, software must be made available to editors and reviewers. We strongly encourage code deposition in a community repository (e.g. GitHub). See the Nature Portfolio guidelines for submitting code & software for further information.

Data

Policy information about availability of data

All manuscripts must include a data availability statement. This statement should provide the following information, where applicable:

- Accession codes, unique identifiers, or web links for publicly available datasets
- A description of any restrictions on data availability
- For clinical datasets or third party data, please ensure that the statement adheres to our policy

The authors declare that all data supporting our findings have been revealed in the main text and figures, and in the supplementary information, or can be obtained from the leading correspondence (L.Z.) upon request.

Human research participants

Policy information about studies involving human research participants and Sex and Gender in Research.

Reporting on sex and gender	N/A
Population characteristics	N/A
Recruitment	N/A
Ethics oversight	N/A

Note that full information on the approval of the study protocol must also be provided in the manuscript.

Field-specific reporting

Please select the one below that is the best fit for your research. If you are not sure, read the appropriate sections before making your selection.

[x] Life sciences [ ] Behavioural & social sciences [ ] Ecological, evolutionary & environmental sciences

For a reference copy of the document with all sections, see nature.com/documents/nr-reporting-summary-flat.pdf

Life sciences study design

All studies must disclose on these points even when the disclosure is negative.

Sample size	The sample size was determined based on literatures from the same field (e.g. Nature 2021:589, 426-430), and in consideration of animal size limit due to animal welfare requirement and animal experimental ethical code.
Data exclusions	No data were excluded.
Replication	All experiments were performed on multiple animals from different litters. The exact N number has been stated in the figure legend.
Randomization	Mice were randomly assigned into each group before the initiation of experiments.
Blinding	All behavioral tests and ex vivo electrophysiological recording (including data analysis) were performed by persons who were blinded to the grouping.

Reporting for specific materials, systems and methods

We require information from authors about some types of materials, experimental systems and methods used in many studies. Here, indicate whether each material, system or method listed is relevant to your study. If you are not sure if a list item applies to your research, read the appropriate section before selecting a response.

Materials & experimental systems

n/a	Involved in the study
[ ]	[x] Antibodies
[x]	[ ] Eukaryotic cell lines
[x]	[ ] Palaeontology and archaeology
[ ]	[x] Animals and other organisms
[x]	[ ] Clinical data
[x]	[ ] Dual use research of concern

Methods

n/a	Involved in the study
[x]	[ ] ChIP-seq
[x]	[ ] Flow cytometry
[x]	[ ] MRI-based neuroimaging

Antibodies

Antibodies used	All antibodies used in this study were listed in Materials and Methods section

Validation	All antibodies used in this study has been extensively used and validated previously, see the list of RRID and reference as follows. Anti-CaMKIIa Antibody. RRID: ab5683.To mark the expression of excitatory neurons in the mPFC brain region of mice after Virus injection, Immunofluorescence was used at a concentration of 1:1000 (https://www.abcam.cn/camkii-alpha-phospho-t286-antibody-ab5683.html)

Animals and other research organisms

Policy information about studies involving animals; ARRIVE guidelines recommended for reporting animal research, and Sex and Gender in Research

Laboratory animals	Male C577Bl/6 mice were purchased from Guangdong Medical Laboratory Animal Center.
Wild animals	N/A
Reporting on sex	N/A
Field-collected samples	N/A
Ethics oversight	All animal experimental protocols were pre-approved by the Ethics Committee of Experimental Animals of Jinan University in accordance with Institutional Animal Care and Use Committee guidelines for animal research.

Note that full information on the approval of the study protocol must also be provided in the manuscript.
